# Permeate Flux Enhancement in Air Gap Membrane Distillation Modules with Inserting Λ-Ribs Carbon-Fiber Open Slots

**DOI:** 10.3390/membranes13010066

**Published:** 2023-01-04

**Authors:** Chii-Dong Ho, Luke Chen, Yan-Ling Yang, Shih-Ting Chen, Jun Wei Lim, Zheng-Zhong Chen

**Affiliations:** 1 Department of Chemical and Materials Engineering, Tamkang University, Tamsui, New Taipei 251, Taiwan; 2 Department of Water Resources and Environmental Engineering, Tamkang University, Tamsui, New Taipei 251, Taiwan; 3 Department of Fundamental and Applied Sciences, HICoE-Centre for Biofuel and Biochemical Research, Institute of Self-Sustainable Building, Universiti Teknologi PETRONAS, Seri Iskandar 32610, Perak Darul Ridzuan, Malaysia

**Keywords:** air gap membrane distillation, permeate flux, hydrodynamic angles, temperature polarization, carbon-fiber open slots

## Abstract

A novel design of an air gap membrane distillation (AGMD) module was proposed to enhance the permeate flux improvement for the desalination of pure water productivity. The modeling equations for predicting permeate flux in the AGMD module by inserting Λ-ribs carbon-fiber open slots under various hydrodynamic angles were developed theoretically and experimentally. The temperature distributions of both hot and cold feed streams were represented graphically with the hot saline flow rate, inlet saline temperature, and carbon-fiber hydrodynamic angles as parameters. The results showed a good agreement between the experimental results and theoretical predictions. Designed by inserting Λ-ribs carbon-fiber open slots into the flow channel, the membrane distillation module was implemented to act as an eddy promoter and yield an augmented turbulence flow. The effect of Λ-ribs carbon-fiber open slots not only assured the membrane stability by preventing vibration but also increased the permeate flux by diminishing the temperature polarization of the thermal boundary layer. The permeate flux improvement by inserting Λ-ribs carbon-fiber open slots in the AGMD module provided the maximum relative increment of up to 15.6% due to the diminution of the concentration polarization effect. The experimental data was incorporated with the hydrodynamic angle of Λ-ribs carbon-fiber open slots to correlate the enhancement factor with the Nusselt numbers to confirm the theoretical predictions. The accuracy derivation between the experimental results and theoretical predictions was pretty good, within 9.95≤E≤1.85. The effects of operating and designing parameters of hot saline flow rate, inlet saline temperature, and hydrodynamic angle on the permeate flux were also delineated by considering both the power consumption increment and permeate flux enhancement.

## 1. Introduction

The membrane distillation (MD) process becomes one of the crucial issues in arid and semi-arid areas of fresh water scarcity [1,2] because of its simplicity and low-energy requirements [3], which makes the MD system an economic feasibility option. The desalination process is where the volatile species in the hot feed stream are vaporized and transported across the porous hydrophobic membrane in order to produce a high-purity liquid [4]. The vapor pressure gradient across the hydrophobic microporous membrane resulting from a trans-membrane temperature variance to drive the vapor flux permeating from the hot side to the cold side of condensates collected [5]. The existence of temperature differences plays a vital review in their features and device performance [6] and attracts global attention with considerable heat losses [7]. The temperature and concentration polarization effects were attributed to significant limits for the MD permeate flux performance and major assessment factors at industrial application scales [8,9]. AGMD is one of the most energy-efficient MD device among different types of MD modules [10], which can be operated in a relatively low temperature and low-pressure stream driven by low-grade heat from renewable energy sources [11,12]. However, the high heat loss builds up the temperature gradient inside the boundary layers of both membrane-surface sides [8] due to the temperature polarization effect with the significant thermal resistance occurrence. Identification of the magnitude of these effects in improving permeate flux enhancement on the MD system was proposed to reduce the polarization effects by using eddy promoters in the flow channel, including various approaches, e.g., spacers [13], filament [14,15], roughened surface [16], turbulence promoter [17], and porous and non-porous hollow fiber module [18]. Mitigation of the thermal boundary layer by adding eddy promoters could generate vortices and secondary flows in the flow stream, and thus, the permeate flux enhancement is achieved accordingly.

A new design, by inserting Λ-ribs carbon-fiber open slots in a flat-plate AGMD module, was developed theoretically and experimentally to improve distillate flux efficiency. The convective heat transfer coefficient and latent heat across the membrane were incorporated into the energy balance equations derived in the present study to solve the temperature fields, which depend on the turbulence intensity augmentation from inserting eddy promoters. However, the pressure drops with power consumption increments have been taken into account in the economic analysis [19] due to implementing eddy promoters in the flow channel for ultrafiltration and direct contact membrane distillation (DCMD) modules [20]. Many studies offer more insight based on mathematical models to develop strategies for improving permeate flux enhancement [21], and the heat and mass transport modeling approaches were reviewed methodically by Dong [22].

The present work focuses on the AGMD module to improve the device performance by inserting Λ-ribs carbon-fiber open slots into the flow channel of AGMD modules, which were found to disrupt the laminar boundary layer and enhance the local shear stress owing to enhancing the local surface shear stress, and thus, yielding augmentation of driving-force temperature gradients comes out with the increment of trans-membrane permeate flux [8,9]. The new construct of the module, by inserting Λ-ribs carbon-fiber open slots in the flow channel, was studied experimentally and numerically with its performance for saline water desalination, and was obtained in terms of a correlated expression [14,23] of Nusselt numbers. The main purpose of this study is to investigate the effects of designing and operating variables on the device performance, and the comparison was made between the modules with empty channels and the improved module with inserting Λ-ribs carbon-fiber open slots under various operating conditions. An alternative strategy for the membrane distillation modules improves the permeate flux by inserting Λ-ribs carbon-fiber open slots, and adjusting the open slots’ hydrodynamic angles in the flow channel of flat-plate AGMD modules, which disturbs the thermal boundary layer and yields a higher permeate flux improvement for enhanced heat and mass transfer. The suitable selection on economic feasibility was identified and explored theoretically by considering both the permeate flux improvement and power consumption increment.

## 2. Experimental Apparatus and Procedure

**Figure 1:**
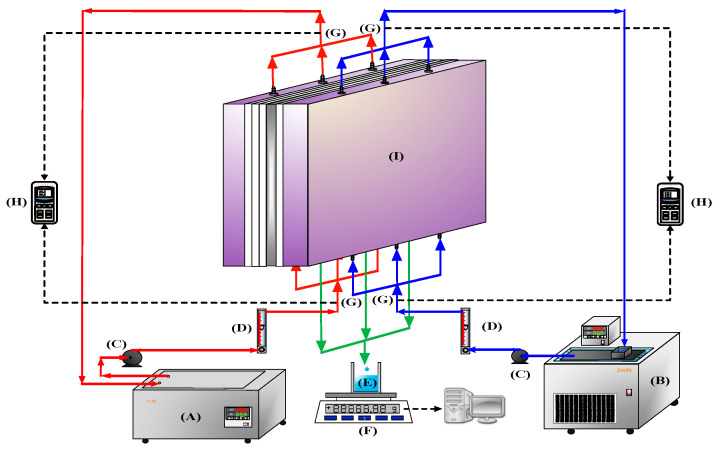
Experimental configuration of the air gap membrane distillation (AGMD) system: (**A**) hot fluid thermostat; (**B**) cold fluid thermostat; (**C**) pump; (**D**) flow meter; (**E**) beaker; (**F**) electronic balance; (**G**) temperature probe; (**H**) temperature indicator; (**I**) the AGMD module.

**Figure 2:**
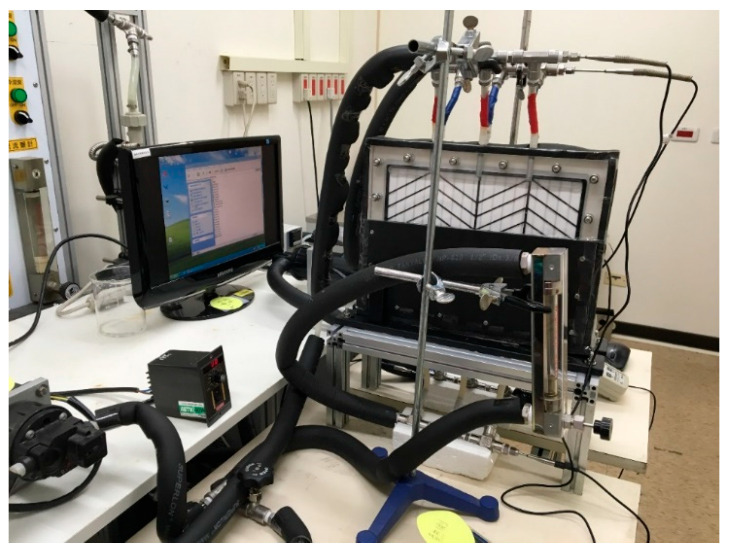
A photo of the experimental apparatus of the AGMD system.

**Figure 3:**
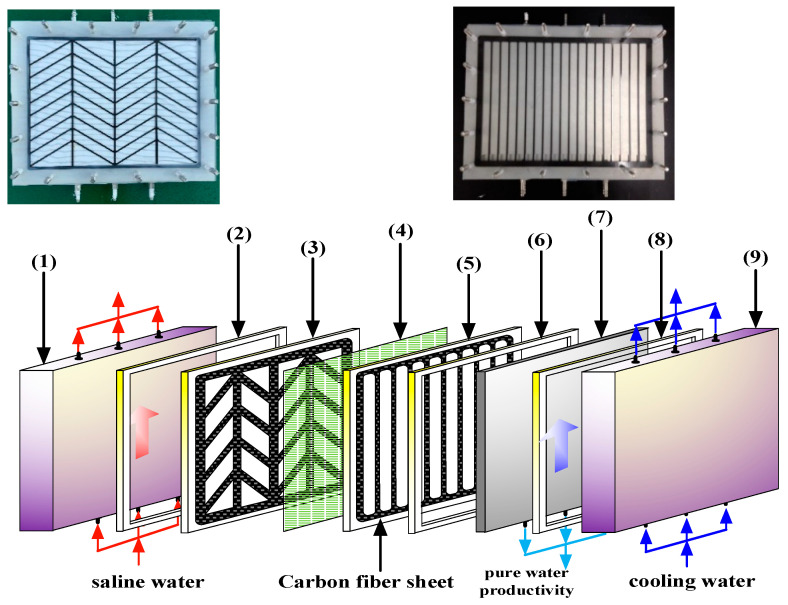
Components of the inserting Λ-ribs carbon-fiber open slots of the AGMD module. (1) Acrylic plate; (2) silicon rubber sealing; (3) Λ -ribs carbon-fiber open slots; (4) PTFE membrane; (5) line carbon-fiber open slots; (6) silicon rubber sealing; (7) aluminum plate; (8) silicon rubber sealing; and (9) acrylic plate.

The experimental setup of the AGMD modules with/without (empty channel) Λ-ribs carbon-fiber open slots is presented schematically in Figure 1, and a photo of the present experimental apparatus is shown in Figure 2 with the acrylic plates as outside walls. The two acrylic plates of the module unit have three holes flowing in and out at both the entrance and exit ends, respectively, and the hot-feed and cold-feed channels are stacked together, as illustrated in Figure 3.

The hot and cold channel’s length, width, and height are 0.21 m, 0.29 m, and 2 mm, respectively. The hot feed saline water of 3.5 wt% NaCl and pure water were transported from two thermostats (G-50, 60 L, 3500 W, DENG YNG, Kaohsiung, Taiwan) through the flat-plate module using conventional pumps (51K40RA-A, ASTK, New Taipei, Taiwan) to regulate steadily two inlet streams at specified temperatures, respectively. The experimental runs were carried out for various hot feed temperatures (40, 45, 50, 55 °C), and both inlet and outlet temperatures were measured using thermometer probes (Type K/J, Tutron, New Taipei, Taiwan) connected to both streams of the flat-plate membrane modules. The permeate flux collected in the cold side was then weighted using an electronic balance (XS 4250C, Precisa Gravimetrics AG, Dietikon, Switerland) and recorded on the PC. The operation conditions of saline feed stream with various flow rates (6.67 × 10^−6^), 8.33 × 10^−6^, 11.7 × 10^−6^ and 15.0 × 10^−6^ m^3^/s) were adjusted by using flow meters (FE-091312-D (hot stream), FN-0423112-F (cold stream), Fong-Jei, Hsinchu, Taiwan) and controller (US-2000-40W, ASTK, New Taipei, Taiwan). Hydrophobic polytetrafluoroethylene (PTFE) membrane (J020A330R, Toyo Roshi Kaisha, Ltd., Tokyo, Japan) supported by polypropylene net (PP) with a nominal pore size of 0.2 μm, a porosity of 0.72, and a thickness of 130 μm was conducted in the present experimental runs. Two hydrodynamic angles and two carbon-fiber open slot’s widths were implemented into flow channels to generate vortices with changing direction along the hot saline water feed flows from the grid to grid, and the line carbon-fiber open slots were used in the permeate side to prevent the vibration of membrane acting as a supporter, as shown in Figure 3. The thermal conductivity of carbon-fiber open slots within 374 and 409 W/mK with negligible thermal resistance as compared to that of copper of 400 W/mK.

## 3. Theoretical Modeling of Mass and Heat Transfer in AGMD Modules

**Figure 4:**
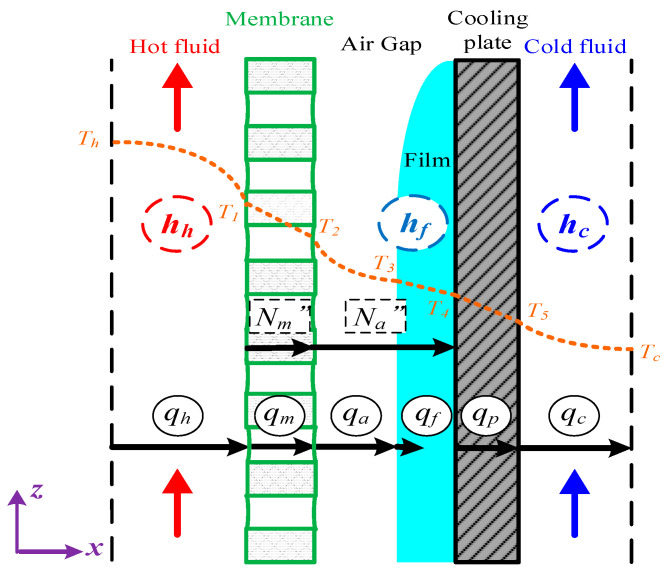
Schematic thermal boundary layers and temperature profiles of the AGMD module.

**Figure 5:**
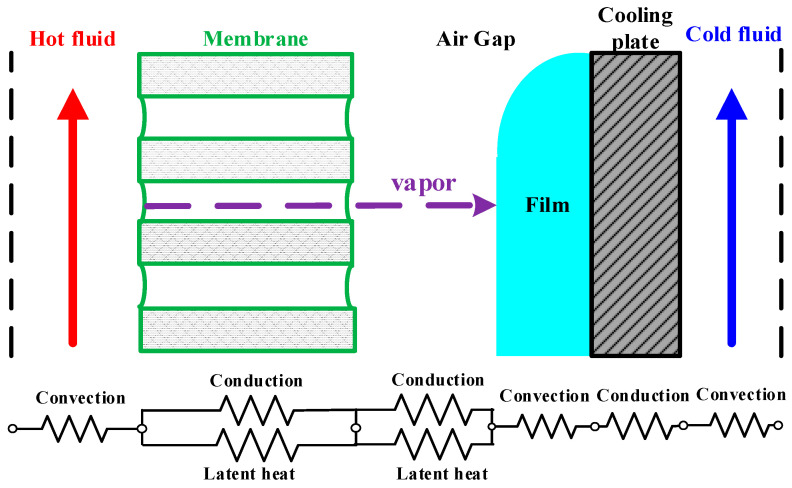
Schematic heat transfer resistances in the AGMD module.

A representation of heat- and mass-transfer models were formulated in the AGMD module and depicted in Figure 4, while the schematic thermal transfer resistances in series are shown in Figure 5.

The permeate flux is dependent on the vapor pressure across the trans-membrane surfaces on the saline feed stream and air gap, as well as the membrane permeation coefficient ($c_{m}$) [24,25] as

(1) $$N^{\prime \prime }=c_{T}\left(P_{1}^{sat}-P_{3}^{sat}\right)=c_{T}{\frac{dP}{dT}|}_{T_{m}}\left(P_{1}^{sat}-P_{3}^{sat}\right)=c_{T}\frac{P_{avg}\lambda M_{w}}{RT_{m}^{2}}\left(P_{1}^{sat}-P_{3}^{sat}\right)$$

where $P_{1}^{sat\;}$ and $P_{3}^{sat\;}$ are the saturated pressure on the membrane surface of the hot stream and the condensate film, respectively, and in which $c_{T}$ is as follows:

(2) $$c_{T}={\left(\frac{1}{c_{m}}+\frac{1}{c_{a}}\right)}^{-1}={\left[{\left[1.064\frac{\varepsilon r}{\tau \delta_{m}}{\left(\frac{M_{w}}{RT_{m}}\right)}^{\frac{1}{2}}\right]}^{-1}+{\left[\frac{1}{{\left|Y_{m}\right|}_{ln}\frac{D_{m}\varepsilon }{\delta_{m}\tau }\frac{M_{w}}{RT_{m}}}\right]}^{-1}+{\left[\frac{1}{{\left|Y_{a}\right|}_{ln}\frac{D_{a}}{\delta_{a}}\frac{M_{w}}{RT_{a}}}\right]}^{-1}\right]}^{-1}$$

where the tortuosity $\tau =\frac{1}{\varepsilon }$ [26].

Theoretical modeling of both heat- and mass-transfer behaviors for a non-isothermal process inside the AGMD module is schematically illustrated in Figure 4. The heat transfer in the AGMD system caused by the temperature gradient across each component of the module. Using the equations for the conservation of enthalpy flow by considering both conduction heat flow and mass diffusion enthalpy flow through the composite system [27] as follows:

(3) $$q^{\prime \prime }=h_{h}\left(T_{h}-T_{1}\right)\;=\frac{k_{m}}{\delta_{m}}\left(T_{1}-T_{2}\right)+N^{\prime \prime }\lambda =\frac{k_{a}}{\delta_{a}}\left(T_{2}-T_{3}\right)+N^{\prime \prime }\lambda =h_{f}\left(T_{3}-T_{4}\right)=\frac{k_{p}}{\delta_{p}}\left(T_{4}-T_{5}\right)=h_{c}\left(T_{5}-T_{c}\right)$$

Equating the energy conservation of the heat flow and latent heat in each region for two intervals, $\left(T_{1}-T_{2}\right)$ and $\left(T_{2}-T_{3}\right)$, yields the total heat transfer coefficient of the hot feed side and the surface of the condensate film as follows:

(4) $$q^{\prime \prime }=\left\{{\left(\frac{\delta_{m}}{k_{m}}+\frac{\delta_{a}}{k_{a}}\right)}^{-1}+\left[c_{T}\frac{\left(\left(1-x_{\mathrm{NaCl}}\right)\left(1-0.5x_{\mathrm{NaCl}}-10x_{\mathrm{NaCl}}^{2}\right)P_{w}+P_{3}\right)\lambda^{2}M_{w}}{2RT_{avg}^{2}}\right]\right\}\left(T_{1}-T_{3}\right)=H_{m}\left(T_{1}-T_{3}\right)$$

in which

(5) $$H_{m}={\left(\frac{\delta_{m}}{k_{m}}+\frac{\delta_{a}}{k_{a}}\right)}^{-1}+\left[c_{T}\frac{\left(\left(1-x_{\mathrm{NaCl}}\right)\left(1-0.5x_{\mathrm{NaCl}}-10x_{\mathrm{NaCl}}^{2}\right)P_{w}+P_{3}\right)\lambda^{2}M_{w}}{2RT_{avg}^{2}}\right]$$

where N”λ is referred to the latent heat of vaporization and $a_{w}=1-0.5x_{\mathrm{NaCl}}-10x_{\mathrm{NaCl}}^{2}$ [4] is the activity coefficient. In addition, the thermal conductivity of the membrane $k_{m}$ can be determined by the thermal conductivities of vapor in the membrane pore $k_{g}$ and the solid membrane material $k_{s}$ by Warner [28] as:

(6) $$k_{m}=\varepsilon \;k_{g}+\left(1-\varepsilon \right)k_{s}$$

Similarly, the overall heat transfer coefficient of the cold feed side was obtained as

(7) $$q^{\prime \prime }={\left(\frac{1}{h_{f}}+\frac{\delta_{p}}{k_{p}}+\frac{1}{h_{c}}\right)}^{-1}\left(T_{3}-T_{c}\right)=H_{c}\left(T_{3}-T_{c}\right)$$

The bulk temperature $T_{h}$ of the hot saline feed stream drops to the membrane surface temperature $T_{1}$, and thus a larger amount of heat was needed to vaporize water at the membrane surface owing to the noticeable temperature polarization effect. The heat transfer rate-limiting step in the thermal boundary layer due to the large difference in temperature between the bulk feed stream and the membrane surface was examined by the temperature polarization coefficient $T_{PC}$, which is an indicator defined as the ratio of membrane surface temperatures’ gradient to bulk temperatures’ gradient and can be expressed in terms of heat transfer coefficients as follows:

(8) $$T_{PC}=\frac{\left(T_{1}-T_{3}\right)}{\left(T_{h}-T_{c}\right)}=\frac{h_{h}H_{c}}{h_{h}H_{c}+h_{h}H_{m}+H_{c}H_{m}}$$

**Figure 6:**
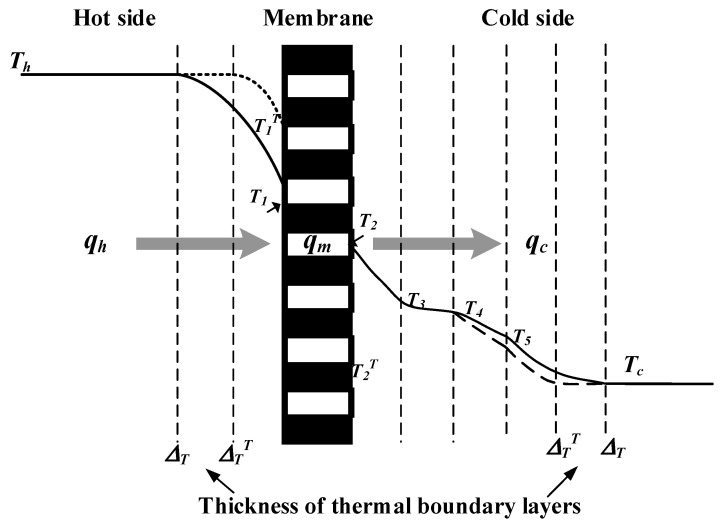
Schematic mass and heat transfer resistances in the AGMD module.

Implementing Λ-ribs carbon-fiber open slots into the hot feed stream reduces the thickness of the thermal boundary layer at the membrane surface, as shown in Figure 6, and thus, $T_{PC\;}$ increases (i.e., the reduction of temperature polarization effect).

**Figure 7:**
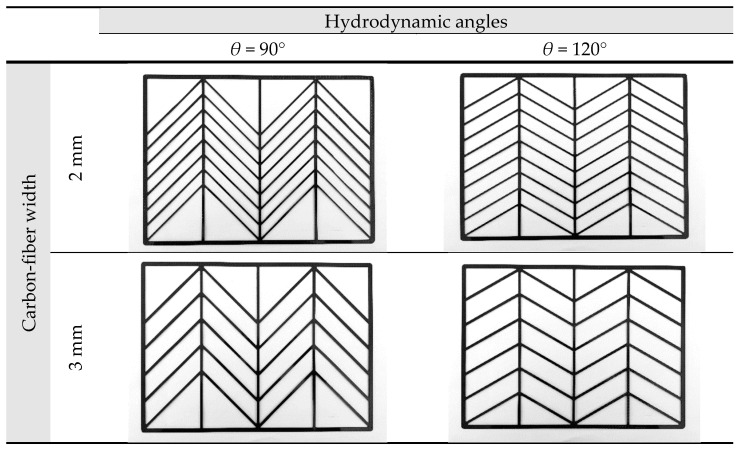
Components of inserting various Λ-ribs hydrodynamic angles and carbon-fiber widths.

**Figure 8:**
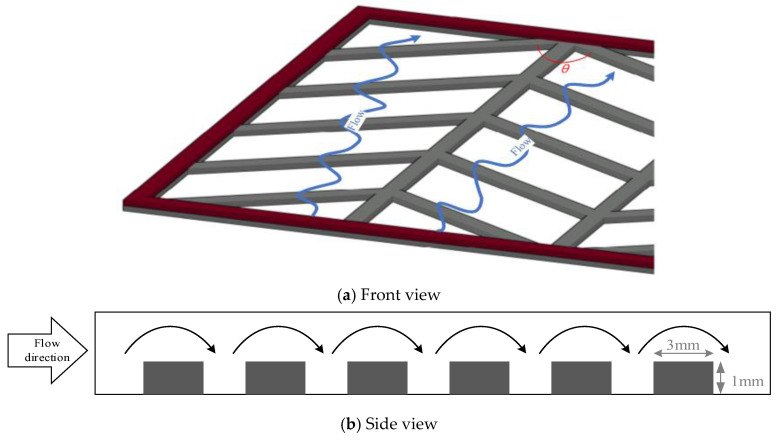
Saline feed flow streamlines in the Λ-ribs carbon-fiber channel with hydrodynamic angles.

The driving force for permeate flux decreases when the temperature gradient at the membrane surface on the hot feed side is less than the bulk temperature gradient. An attempt was proposed in the last two decades to augment turbulence intensity by inserting eddy promoters into the flow channel to enlarge the turbulence intensity. Restated, the reduction of the temperature polarization effect in decreasing the temperature gradient between the bulk stream and membrane surface indicates that the Λ-ribs carbon-fiber open slots could disturb the thermal boundary layer to strengthen a larger convective heat-transfer coefficient. It comes out with a thinner thermal boundary layer owing to increasing the larger $T_{PC}$ value, and thus, a higher permeate flux is achieved. Various hydrodynamic angles and carbon-fiber widths inserted into the flowing and empty channels (without inserting carbon fiber) were conducted in the experimental work under the same total coverage area, as shown in Figure 7, respectively. A proportion of the hot saline water feed flows across over the carbon-fiber filaments from the open slot to another open slot, and the direction change induced by the hydrodynamic angle due to following a zigzag-like pathway, as seen in Figure 8.

**Figure 9:**
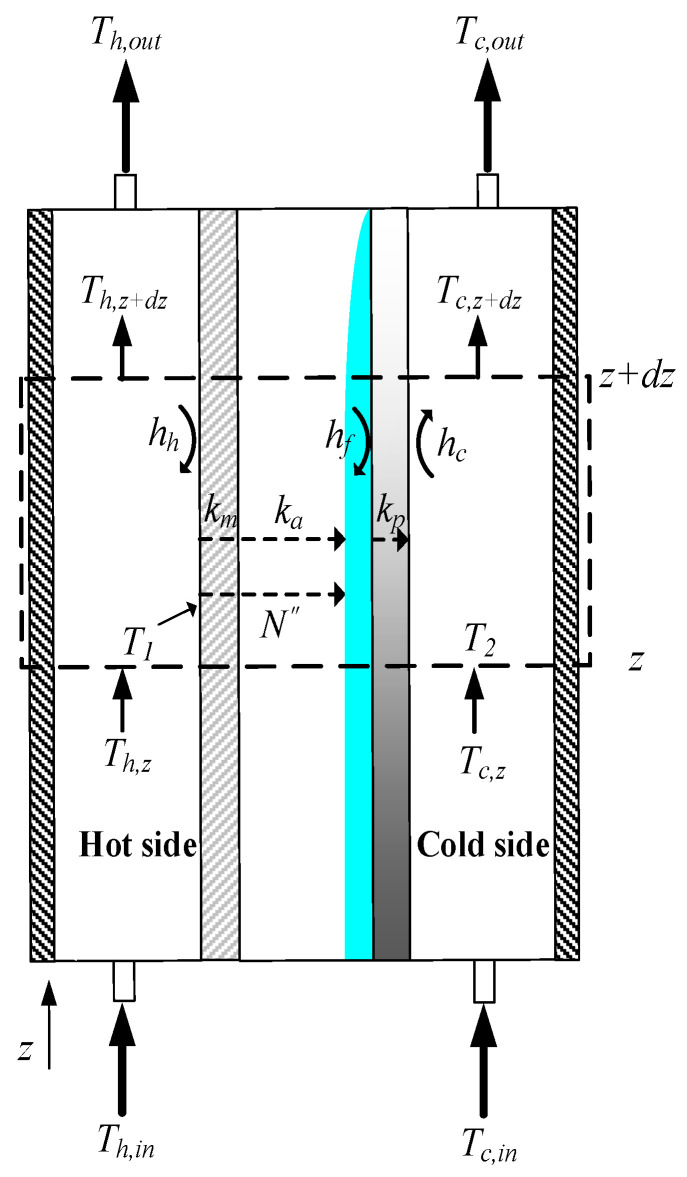
The energy balance made within a finite fluid element.

The longitudinal temperature distributions of both hot and cold feed streams were estimated by the one-dimensional modeling equations from the heat balance at steady state, which is presented in a finite fluid element of the energy-flow diagram, as shown in Figure 9.

(9) $$\frac{dT_{h}}{dz}=\frac{-q^{\prime \prime }W}{Q_{h}\;\rho_{h}C_{p,h}}=\frac{-W}{Q_{h}\;\rho_{h}C_{p,h}}H_{m}T_{PC}\left(T_{h}-T_{c}\right)$$

(10) $$\frac{dT_{c}}{dz}=\frac{q^{\prime \prime }W}{Q_{c}\;\rho_{c}C_{p,c}}=\frac{W}{Q_{c}\;\rho_{c}C_{p,c}}H_{m}T_{PC}\left(T_{h}-T_{c}\right)$$

**Figure 10:**
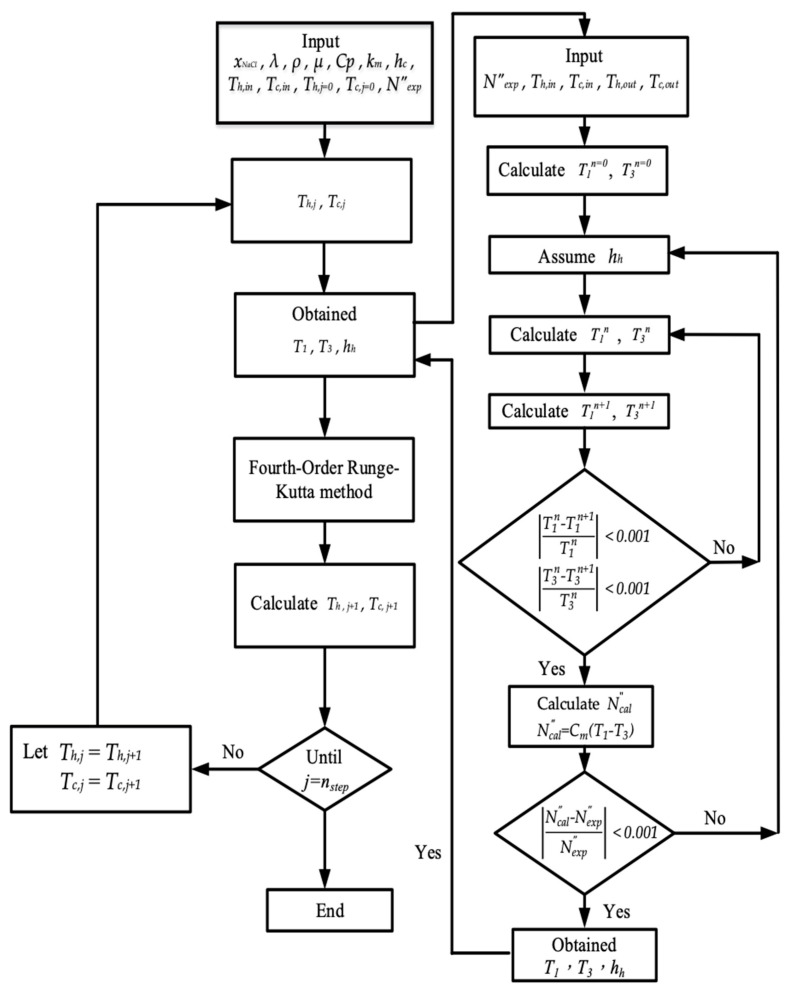
Flow chart for solving membrane surface temperatures and heat transfer coefficients.

The temperature distributions of both hot and cold feed streams were solved in the above two simultaneous first-order ordinary differential equations of Equations (9) and (10) with the use of the estimated convective heat-transfer coefficients and calculated iteratively of Figure 10 by marching the fourth-order Runge–Kutta method numerically along the length of the AGMD module. By following the similar flowchart of calculation procedure adapted from [29], and thus, the theoretical predictions of permeate fluxes and the permeate flux enhancement were obtained accordingly. The temperature distributions were predicted theoretically not only in the hot/cold bulk flows ($T_{h}$ and $T_{c}$) but also on the membrane surfaces ($T_{1}$ and $T_{3}$) of both hot and cold feed streams, respectively. Meanwhile, the convective heat-transfer coefficients were calculated and validated by the experimental results. Experimental runs were carried out in this study to demonstrate the device performances of permeate fluxes by using the module with inserting Λ-ribs carbon-fiber widths in flow channels and the module using the empty channel.

## 4. Permeate Flux Enhancement and Power Consumption Increment

The degree of heat transfer enhancement is commonly expressed by an enhancement factor, $\alpha^{E}$, which is the ratio of the heat transfer coefficients of the improved module by inserting Λ-ribs carbon-fiber open slots to that of the empty channel (without inserting carbon fiber). The Λ-ribs carbon-fiber open slots inserted in the conduit of the hot feed stream are implemented as compared to the module of the empty channel, and the permeate flux enhancement factor $\alpha^{E}$ depends on the hydrodynamic angles and carbon-fiber widths [30] with respect to the characterization of the turbulence intensity as follows:

(11) $$\alpha^{E}=\frac{Nu^{E}}{Nu_{lam}}$$

where

(12) $$Nu^{E}=\frac{h_{h}D_{h,h}}{k}\;\mathrm{for}\;a\;\mathrm{module}\;\mathrm{inserting}\;\Lambda -\mathrm{ribs}\;\mathrm{carbon}-\mathrm{fiber}\;\mathrm{open}\;\mathrm{slots}$$

(13) $$Nu_{lam}=4.36+\frac{0.036RePr\left(\frac{D_{h,h}}{L}\right)}{1+0.011{\left[RePr\left(\frac{D_{h,h}}{L}\right)\right]}^{0.8}}\;\mathrm{for}\;\mathrm{the}\;\mathrm{laminar}\;\mathrm{flow}\;\mathrm{in}\;a\;\mathrm{flat}\;\mathrm{membrane}\;\mathrm{module}.$$

Applying the regressed correlation approach and quantifying the enhanced permeate flux due to the eddy promoter of the module were solved directly through the permeate flux enhancement factor, which was based on dimensional analysis of Buckingham’s π theorem. Thus, the Nusselt number can be related to dimensionless groups to lump the influence of eddies and vortices created by the turbulent flow due to implementing the Λ-ribs carbon-fiber open slots as:

(14) $$Nu^{E}=f\;\left(\frac{W_{e}}{D_{h,h}},\sin\;\theta \right)$$

where $W_{e}$ and $D_{h,h}$ are the Λ-ribs carbon-fiber width and hydrodynamic angles of the hot stream-side, respectively.

The power consumption increment is required due to inserting Λ-ribs carbon-fiber open slots into the saline feed channel. The friction losses were calculated in determining the power consumption by using the Fanning friction factor $f_{F}$ [31]:

(15) $$H_{i}={\dot{m}}_{h}\ell w_{f,h}+{\dot{m}}_{c}\ell w_{f,c}=Q_{h}\rho_{h}\ell w_{f,h}+Q_{c}\rho_{c}\ell w_{f,c}i=carbon\;fiber,\;empty$$

(16) $$\ell w_{f,j}=\frac{2f_{F,j}{\overset{¯}{v}}_{j}^{2}L}{D_{h,i}},\;j=h,c$$

in which ($\beta =\frac{d}{W}$) [32]:

(17) fF,j=24(1−1.3553 β+1.9467β 2−1.7012β 3+0.9564β 4−0.2537β 5)Re j, j=h, c.

The power consumption increment $I_{P}$ due to the friction losses in the conduits can be readily derived as follows:

(18) $$I_{p}=\frac{H_{carbon\;fiber}-H_{empty}}{H_{empty}}\times 100\%.$$

## 5. Results and Discussion

**Figure 11:**
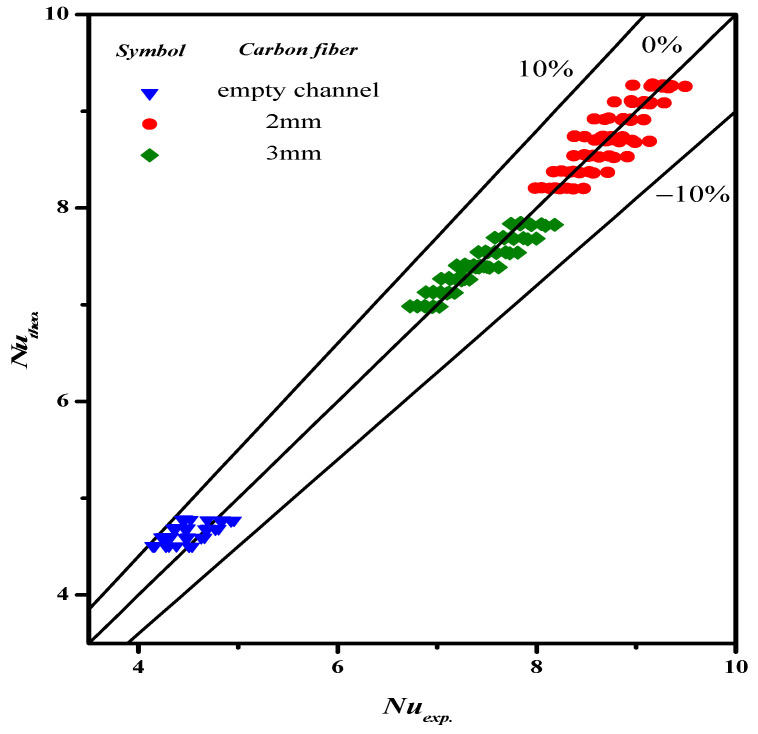
Comparisons of both calculated and experimental Nusselt numbers.

The experimental data with empty channel (without implementing carbon-fiber open slots instead of using nylon fiber) and 2 mm and 3 mm Λ-ribs carbon-fiber widths were used to determine the correlation for the permeate enhancement factor $\alpha^{E}$, as expressed in Equation (19), which was regressed by setting up the normal equations for the least square parameters. The resultant regression analysis from curve-fitting with the squared correlation coefficient ($R^{2}=0.951$), as shown in Figure 11.

(19) $$Nu^{E}=1.527{\left(\frac{W_{e}}{D_{h}}\right)}^{-0.427}{\left(Sin\theta \right)}^{0.504}$$

**Figure 12:**
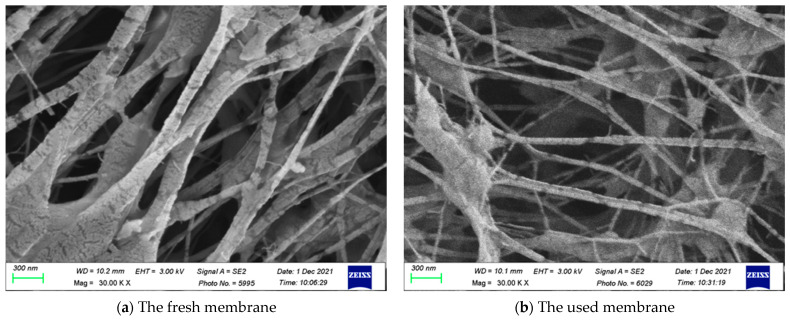
SEM micrographs of the fresh and used membranes of experimental runs.

Figure 12 shows the SEM micrographs of the fresh and used membranes of experimental runs. The SEM images indicated that the presence of Λ-ribs carbon-fiber open slots would not be a problem of fouling or scaling in conducting experimental runs.

**Figure 13:**
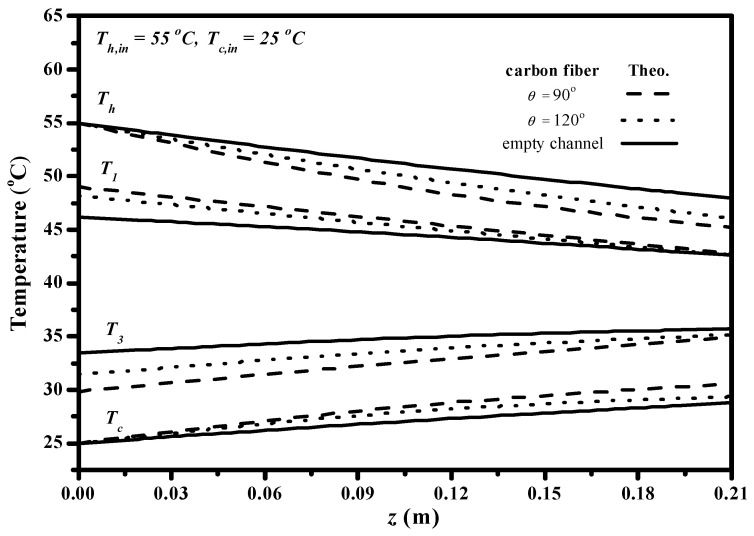
The effect of various hydrodynamic angles with a 2 mm carbon-fiber width on temperature profiles ($Q_{h}=8.33\times 10^{-6}\frac{m^{3}}{s},\;Q_{c}=1.5\times 10^{-5}\frac{m^{3}}{s}$).

Implementing carbon-fiber open slots play an important role in the permeate flux improvement due to disturbing the flow stream inside the concentration boundary layer, and thus, the higher turbulence intensity was augmented owing to reducing heat-transfer resistances. The effect of the carbon-fiber open slots in the AGMD module on the longitudinal temperature profiles of both hot and cold feed streams is shown in Figure 13, in which the temperature gradient tapered from the higher value at the entrance to the outlet streams. The temperature profiles show that temperature deviations (say temperature gradients between the membrane surface temperature and bulk temperature of both hot and cold feed streams) are considerably decreased while the AGMD modules with inserting Λ-ribs carbon-fiber open slots for both $120^{{}^{\circ}}$ and $90^{{}^{\circ}}$ hydrodynamic angles as compared to the module using the empty channel. The temperature polarization effect was reduced for a promising result by inserting Λ-ribs carbon-fiber open slots in the flow channel due to eddy turbulence increment and investigated by computational simulation [33]. One can find that the temperature gradient of inserting Λ-ribs carbon-fiber open slots of the $90^{{}^{\circ}}$ hydrodynamic angle was higher than that of the $120^{{}^{\circ}}$ hydrodynamic angle.

**Figure 14:**
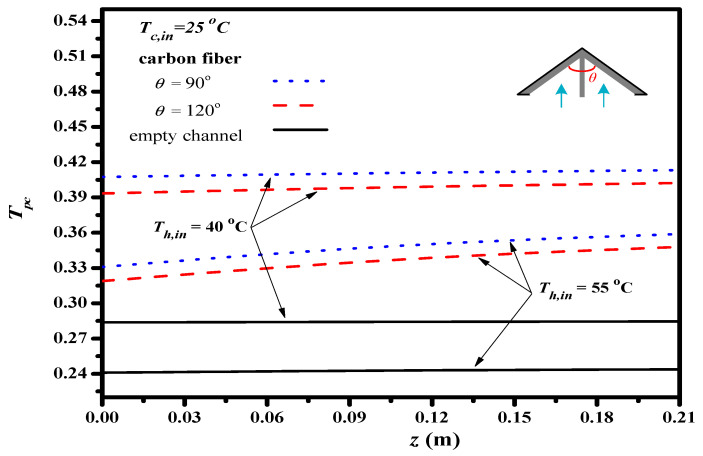
The effect of various hydrodynamic angles of 2 mm carbon-fiber width on $T_{PC\;}$.

The temperature polarization coefficients $T_{PC}$ is defined in Equation (8) and calculated by both feed stream temperature distributions, the results investigated and compared the effects of hydrodynamic angles on the temperature polarization effect, as depicted in Figure 14. Inserting Λ-ribs carbon-fiber open slots of the $90^{{}^{\circ}}$ hydrodynamic angle was found to be a larger $T_{PC}$ value (a higher heat transfer rate) than those of the Λ-ribs carbon-fiber open slots with the $120^{{}^{\circ}}$ hydrodynamic angle and the empty channel as well; this is attributed to more carbon-fiber open slots to strengthen turbulence intensity and shrink the thermal boundary layer with a larger $T_{PC}$ value, and thus, comes out with a higher permeate flux through the hydrophobic membrane. The theoretical results show that a higher inlet temperature of the hot saline feed stream results in a lower $T_{PC}$ values owing to the higher permeate flux associated with the more latent heat of vaporization, $T_{PC}$ increased with a decreasing hydrodynamic angle as well. Improved devices with implementing Λ-ribs carbon-fiber open slots into the hot feed stream could use thinner the thermal boundary layer on the membrane surface due to the strengthened turbulence intensity, which comes out of a reduction of the temperature polarization effect, and thus, the heat transfer enhancements were achieved as compared to the module with using the empty channel.

The good agreement of the theoretical predictions with those obtained from experimental results was achieved. The accuracy deviation [34] of the experimental results from the theoretical predictions was calculated using the following definition as:

(20) $$E\left(\%\right)=\frac{1}{N_{exp}}\overset{N_{exp}}{\underset{i=1}{\sum }}\frac{\left|N_{theo}^{”}-N_{exp}^{”}\right|}{N_{exp}^{”}}\times 100$$

where $N_{exp}$, $N_{theo}^{”}$, and $N_{exp}^{”}$ are the number of experimental runs, theoretical predictions, and experimental results of the permeate fluxes, respectively. The accuracy deviations with two Λ-ribs carbon-fiber widths and two hydrodynamic angles were calculated; the agreement of experimental results deviated from theoretical predictions was pretty good, being within 9.95≤E≤1.85.

**Figure 15:**
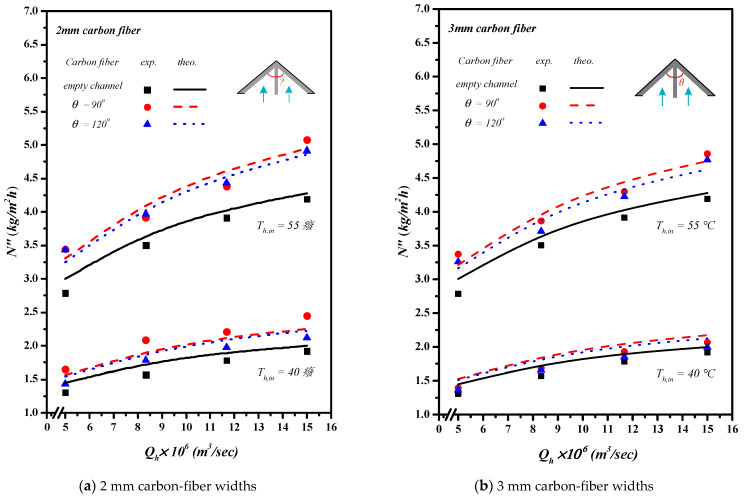
Effects of various hydrodynamic angles and carbon-fiber widths on permeate fluxes.

Inserting Λ-ribs carbon-fiber open slots with two carbon-fiber widths and two hydrodynamic angles produced a higher turbulence intensity that resulted in a higher heat transfer rate, as well as a higher permeate flux. Comparisons were made on theoretical predictions and experimental results of permeate fluxes between the empty channel and the channels by inserting Λ-ribs carbon-fiber open slots of two carbon-fiber widths, say 2 mm and 3 mm, as shown in Figure 15a,b. In general, inserting Λ-ribs carbon-fiber open slots in the AGMD module is a more significant permeate transporting flux through the hydrophobic membrane with the smaller carbon-fiber widths and smaller hydrodynamic angle due to yielding a larger temperature driving-force gradient. Meanwhile, the heat transfer resistance dominating the permeate flux decreased with the increasing inlet volumetric flow rate and inlet saline feed temperature.

**Figure 16:**
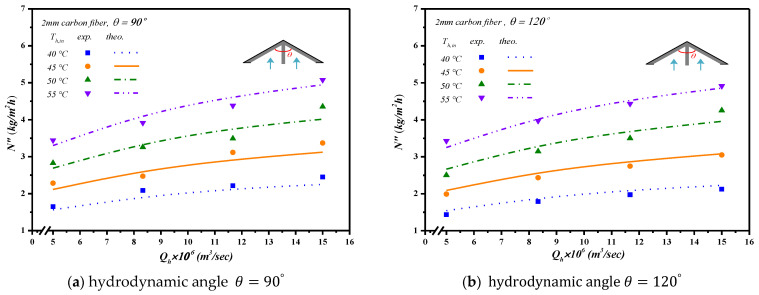
The effects of inlet saline feed temperatures and hydrodynamic angles on permeate fluxes with inserting 2 mm carbon-fiber widths.

**Figure 17:**
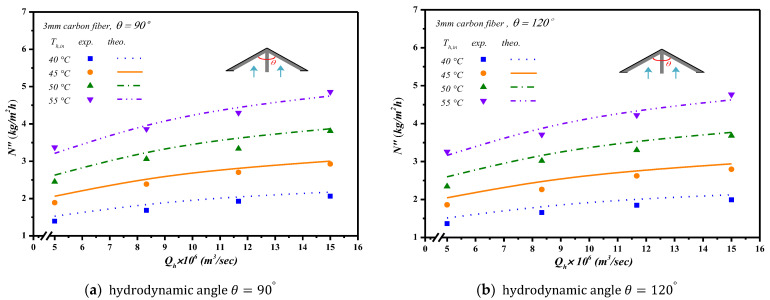
The effects of inlet saline feed temperatures and hydrodynamic angles on permeate fluxes with inserting 3 mm carbon-fiber widths.

The theoretical predictions and experimental results of permeate flux were presented graphically in Figure 16 and Figure 17 for the empty channel and the channels with inserting 2 mm and 3 mm Λ-ribs carbon-fiber open slots under various inlet saline feed temperatures and hydrodynamic angles, respectively. The results show that the permeate flux increased with the saline volumetric flow rate and inlet saline feed temperature but with decreasing hydrodynamic angle and carbon-fiber width.

A percentage increment of permeate flux improvement $I_{E}$ employing a module with inserting Λ-ribs carbon-fiber open slots is best illustrated by calculating in comparisons of the module using the empty channel, as

(21) $$I_{E}=\frac{N_{carbon\;fiber}^{”}-N_{empty}^{”}}{N_{empty}^{”}}\times 100\%$$

The permeate flux enhancements $I_{E}$ of the module with inserting Λ-ribs carbon-fiber open slots into the saline feed stream were significantly achieved in Table 1 for 2 mm and 3 mm carbon-fiber widths, respectively. The theoretical predictions were conducted in comparisons with various hydrodynamic angles, carbon-fiber widths, inlet saline feed temperatures, and inlet volumetric flow rate as parameters. The theoretical predictions show that the permeate flux improvement up to 15.6% is obtained by inserting 2 mm Λ-ribs carbon-fiber open slots as compared to that in the empty channel device, as seen in Table 1. The extent of permeate flux increment is more significant in the smaller hydrodynamic angle and the higher inlet saline feed temperature. Overall, the performance of permeate flux is enhanced by inserting Λ-ribs carbon-fiber open slots into saline feed channel, which plays the important role of eddy promoters in the pressure-driven membrane distillation processes and water treatment technologies.

**Figure 18:**
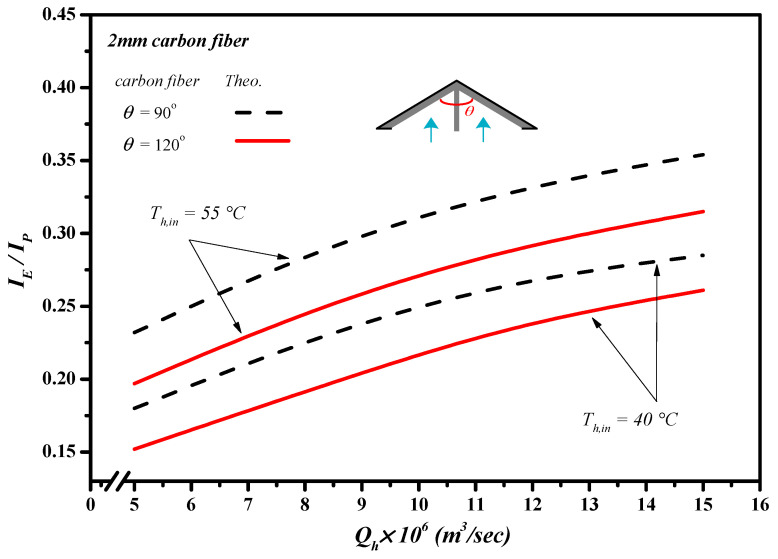
The effects of hydrodynamic angles and inlet saline feed temperatures on the value of $I_{E}/I_{P}$.

Although inserting Λ-ribs carbon-fiber open slots into the flow channel of AGMD modules could create resistance for the feed circulation resulting in the power consumption increment, the permeate flux enhancement was achieved, as well as due to enhancing the local surface shear stress with the increment of trans-membrane permeate flux. This study further examines the permeate flux performance by evaluating the desirable permeate flux increment to the undesirable friction loss increment, say $I_{E}/I_{P}$, due to inserting Λ-ribs carbon-fiber open slots in making the suitable selection from an economic viewpoint. The effects of carbon-fiber widths, hydrodynamic angles, inlet saline feed temperatures, and saline volumetric flow rates are shown in Figure 18 and Table 2, as referred to the ratio of $I_{E}/I_{P}$.

The value of $I_{E}/I_{P}$ increased with increasing saline volumetric flow rate, which indicates that the expenses of energy consumption increment could compensate by the permeate flux enhancement due to utilizing the driving-force temperature gradient more effectively. Furthermore, the ratio of $I_{E}/I_{P}$ of the module with an inserting 2 mm carbon-fiber width was higher than that of the channel with an inserting 3 mm carbon-fiber width consistent with the trend of the permeate flux obtained with the smaller carbon-fiber widths and smaller hydrodynamic angle, and presented in Table 2. Meanwhile, the increase in the saline volumetric flow rate yields a higher ratio of $I_{E}/I_{P}$, and the order of the ratio of $I_{E}/I_{P}$ was expected, with the same trend of the permeate flux.

## 6. Conclusions

There are two innovation points in this module design, which are easy fabricating of Λ-ribs carbon-fiber open slots and a larger ratio of permeate flux increment to power consumption increment. This is the value of the present study. Two hydrodynamic angles of turbulence promoters were used for the AGMD module and compared to an empty channel under two carbon-fiber widths in the present study. Increasing shear stress in the thermal boundary layer on the membrane surface due to inserting turbulence promoters could considerably reduce the temperature polarization effect. The results show that the permeate flux increases with both saline feed flow rate and inlet saline feed temperature, and demonstrate its feasibility of permeate flux enhancement up to 15.6% by inserting Λ-ribs carbon-fiber open slots in the flow channel of the AGMD system. The comparisons of the permeate flux enhancement with inserting turbulence promoters under various carbon-fiber widths and hydrodynamic angles on the permeate flux in AGMD modules were drawn to the following conclusions:

1. The permeate flux increases with decreasing hydrodynamic angle and carbon-fiber width in the AGMD module by inserting Λ-ribs carbon-fiber open slots.
2. The permeate flux enhancement increases with the increase of the volumetric flow rate by inserting Λ-ribs carbon-fiber open slots where the enhancement of the AGMD module using 2 mm open slots is higher than that of the 3 mm one.
3. The higher the inlet saline feed temperature yields, the higher the permeate flux enhancement.
4. A higher permeate flux enhancement was found in the module with inserting Λ-ribs carbon-fiber open slots compared to that in the empty-channel module under both the smaller hydrodynamic angle and carbon-fiber width.
5. The higher ratio of $I_{E}/I_{P}$ was achieved for the module by inserting Λ-ribs carbon-fiber open slots, and was operated under both the higher saline feed flow rate and inlet saline feed temperature, and both the smaller carbon-fiber width and hydrodynamic angle.

A new design in this study includes the advantage of this membrane distillation module in strengthening the turbulence intensity as an alternative strategy for the permeate flux in the AGMD module. The alternative configurations of inserting Λ-ribs carbon-fiber open slots showed a great potential to considerably diminish the temperature polarization effect, and thus the permeate flux was enhanced.

## Figures and Tables

**Table 1:** The effects of hydrodynamic angles and carbon-fiber widths on permeate flux enhancement.

$T_{h,\;in}({}^{\circ}C)$	$Q_{h}\times 10^{6}(m^{3}\;s{-}^{1})$	Empty Channel	2 mm				3 mm			
			$90^{{}^{\circ}}$		$120^{{}^{\circ}}$		$90^{{}^{\circ}}$		$120^{{}^{\circ}}$	
		$N_{theo}^{\prime \prime }\times 10^{3}\mathrm{kg}\;m{-}^{2}\;s{-}^{1}$	$N_{theo}^{\prime \prime }\times 10^{3}\mathrm{kg}\;m{-}^{2}\;s{-}^{1}$	$I_{E}$	$N_{theo}^{\prime \prime }\times 10^{3}\mathrm{kg}\;m{-}^{2}\;s{-}^{1}$	$I_{E}$	$N_{theo}^{\prime \prime }\times 10^{3}\mathrm{kg}\;m{-}^{2}\;s{-}^{1}$	$I_{E}$	$N_{theo}^{\prime \prime }\times 10^{3}\mathrm{kg}\;m{-}^{2}\;s{-}^{1}$	$I_{E}$
40	6.67	0.40	0.43	7.80	0.43	6.42	0.42	5.37	0.42	4.35
	8.33	0.48	0.53	10.2	0.52	8.40	0.52	6.85	0.51	5.11
	11.7	0.53	0.59	11.9	0.58	10.3	0.57	8.01	0.56	5.94
	15.0	0.55	0.63	12.7	0.62	11.4	0.60	8.77	0.59	6.35
45	6.67	0.54	0.59	8.28	0.58	7.07	0.57	5.66	0.57	4.77
	8.33	0.66	0.73	10.7	0.72	9.19	0.71	7.66	0.70	5.62
	11.7	0.72	0.81	12.7	0.80	11.0	0.78	8.91	0.77	6.66
	15.0	0.76	0.87	13.9	0.86	12.4	0.83	9.48	0.82	7.20
50	6.67	0.69	0.75	8.74	0.74	7.76	0.73	6.26	0.72	5.00
	8.33	0.84	0.94	11.5	0.92	10.1	0.91	8.56	0.89	6.09
	11.7	0.92	1.05	14.0	1.03	11.8	1.01	10.0	0.99	7.21
	15.0	0.97	1.12	14.8	1.10	13.1	1.08	10.6	1.05	7.68
55	6.67	0.83	0.92	9.93	0.90	8.23	0.89	6.96	0.88	5.30
	8.33	1.02	1.15	12.7	1.13	10.6	1.12	9.02	1.09	6.68
	11.7	1.12	1.29	14.6	1.26	12.4	1.24	10.5	1.21	7.72
	15.0	1.19	1.37	15.6	1.35	13.6	1.32	11.0	1.29	8.16

**Table 2:** The effects of hydrodynamic angles and carbon-fiber widths on $I_{E}/I_{P}$.

$T_{h,\;in}$ (∘C)	$Q_{h}\times 10^{6}(m^{3}\;s{-}^{1})$	2 mm		3 mm	
		$90^{{}^{\circ}}$	$120^{{}^{\circ}}$	$90^{{}^{\circ}}$	$120^{{}^{\circ}}$
		$I_{E}/I_{P}$	$I_{E}/I_{P}$	$I_{E}/I_{P}$	$I_{E}/I_{P}$
40	6.67	0.18	0.15	0.13	0.11
	8.33	0.23	0.20	0.16	0.13
	11.7	0.27	0.24	0.19	0.15
	15.0	0.29	0.26	0.21	0.16
45	6.67	0.19	0.17	0.14	0.12
	8.33	0.25	0.22	0.18	0.14
	11.7	0.29	0.26	0.21	0.17
	15.0	0.31	0.28	0.22	0.18
50	6.67	0.20	0.19	0.15	0.13
	8.33	0.27	0.24	0.20	0.15
	11.7	0.32	0.28	0.24	0.18
	15.0	0.33	0.30	0.25	0.19
55	6.67	0.23	0.20	0.17	0.14
	8.33	0.29	0.25	0.22	0.17
	11.7	0.33	0.29	0.25	0.19
	15.0	0.35	0.32	0.26	0.20

## Data Availability

Data are contained within the article.

## Abbreviations

A	membrane area (m^2^)
$a_{w}$	water activity in NaCl solution
Cp,c	heat capacity of cold fluid (J kg^−1^K^−1^)
Cp,h	heat capacity of hot fluid (J kg^−1^K^−1^)
$c_{a}$	mass transfer coefficient of air gap region (kg m^−2^ Pa^−1^ s^−1^)
$c_{m}$	mass transfer coefficient of membrane (kg m^−2^ Pa^−1^ s^−1^)
$c_{T}$	overall mass transfer coefficient of membrane (kg m^−2^ Pa^−1^ s^−1^)
d	thickness of empty channel (m)
D	equivalent hydraulic diameter of empty channel (m)
$D_{h,h}$	equivalent hydraulic diameter of hot side (m)
$D_{h,c}$	equivalent hydraulic diameter of cold side (m)
*E*	deviation of experimental results from the theoretical predictions
$f_{F}$	Fanning friction factor
$h_{c}$	convection coefficient of cold stream (W m^−2^ K^−1^)
$h_{h}$	convection coefficient of saline stream (W m^−2^ K^−1^)
$H_{i}$	hydraulic dissipate energy (J kg^−1^), i=promoter,empty
$H_{m}$	thermal convection coefficient of membrane (W m^−2^ K^−1^)
$I_{E}$	increased percentage of permeate flux
$I_{P}$	raised percentage of hydraulic loss
L	axial distance (m)
$k_{a}$	thermal conductivity of the air gap (W m^−1^ K^−1^)
$k_{m}$	thermal conductivity of the membrane (W m^−1^ K^−1^)
$k_{f}$	thermal conductivity coefficient of aqueous solution (W m^−1^ K^−1^)
$k_{g}$	thermal conductivity coefficient of the vapor in the membrane pore (W m^−1^ K^−1^)
$k_{s}$	thermal conductivity coefficient of the solid membrane material (W m^−1^ K^−1^)
$k_{m}$	thermal conductivity of the membrane (W m^−1^ K^−1^)
$k_{p}$	thermal conductivity of cooling plate (W m^−1^ K^−1^)
$\ell w_{f}$	friction loss of conduits (J kg^−1^)
$M_{W}$	molecular weight of water (kg mol^−1^)
$\dot{m}$	mass flow rate (kg s^−1^)
$N^{\prime \prime }$	permeate flux (kg m^−2^ s^−1^)
Nu	Nusselt number
$Nu^{E}$	enhanced Nusselt number with inserting turbulence promoter
$Nu_{lam}$	Nusselt number for laminar flow
P	pressure (Pa)
$P_{1}^{sat}$	saturation vapor pressure in the saline feed stream (Pa)
$P_{3}^{sat}$	saturation vapor pressure in the hot feed flow side (Pa)
$P_{w}{}^{sat}$	saturated vapor pressure of pure water (Pa)
Pr	Prandtl number
q	heat transfer flux (W)
$q_{}^{\prime \prime }$	heat transfer rate (W/m^2^)
Q	volumetric flow rate (m^3^ s^−1^)
R	gas constant (8.314 J mol^−1^ K^−1^)
Re	Reynolds number
r	radius of membrane pore (m)
T	temperature (°C)
$T_{m}$	mean temperature in membrane (°C)
$T_{PC}$	temperature polarization coefficients
$\overset{¯}{v}$	average velocity (m s^−1^)
We	Λ-ribs carbon-fiber width
${\left|Y_{m}\right|}_{\ell n}$	natural log mean mole fraction of air
$x_{NaCl}$	liquid mole fraction of NaCl
$x_{w}$	liquid mole fraction of water
z	axial coordinate along the flow direction (m)
Greek letters	
$\alpha^{E}$	heat-transfer enhancement factor
β	aspect ratio of the channel
ΔP	vapor pressure difference of membrane (Pa)
$\delta_{m}$	thickness of membrane (µm)
ε	membrane porosity
$\varepsilon_{e}$	channel voidage
λ	latent heat of water (J/kg)
μ	fluid viscosity (kg s^−1^ m^−1^)
ρ	density (kg m^−3^)
$T_{PC}$	temperature polarization coefficients
Subscripts	
1	on membrane surface in cold feed side
2	on membrane surface in hot feed side
*a*	in the air gap
*c*	in the cold feed flow channel
*f*	in the condensate film
*h*	in the hot feed flow channel
*m*	in the membrane
*p*	in the cooling plate
*carbon fiber*	inserting Λ-ribs carbon-fiber open slots
*empty*	module with inserting nylon fiber as supporters
*exp*	experimental results
*in*	at the inlet
*lam*	module with empty channel
*out*	at the outlet
*theo*	theoretical predictions
Superscripts	
*E*	the channel with inserting Λ-ribs carbon-fiber open slots
